# The Therapeutics of Whooping Cough

**Published:** 1901-10

**Authors:** 


### The Therapeutics of Whooping Cough.

Dr. Thos. J. Mays, in the September 7th number of the *New York Medical Journal,* has a short article on this subject. He says that unquestionably whooping cough is a spasmodic affection of the pneumogastric nerves due to affinity of a special virus for these structures. In conformity with this belief he has applied counter-irritation with signal benefit. The exact method of applying the treatment is as follows: "Trace the pulsating carotid from behind the angle of the lower jaw to the clavicle on both sides of the neck. This will be a landmark for finding the pneumogastric nerves, which lie in close proximity and slightly behind the carotids. Gentle massage and kneading of this region of the neck, every hour or two, yield beneficial effects in many cases. The application of a strip of mustard plaster from the angle of the lower jaw to the clavicle on each side of the neck, two or three times a day, is almost sure to cause amelioration of the spasmodic cough." Painting over the same region with tincture of iodine or with equal parts of gum camphor, chloral hydrate and menthol also yields good results. As a last resort in bad cases the deep hypodermic injection of six minims of a two and one-half per cent. solution of silver nitrate may be tried.

